# Hemophagocytic Lymphohistiocytosis Secondary to Non-Hodgkin Lymphoma: A Case Report

**DOI:** 10.7759/cureus.77492

**Published:** 2025-01-15

**Authors:** Joana Castro Vieira, Ricardo Madeira, Mafalda Maria Santos, João Vieira Afonso, Ana Cristina Teotónio

**Affiliations:** 1 Internal Medicine, Unidade Local de Saúde do Oeste - Hospital Distrital das Caldas da Rainha, Caldas da Rainha, PRT; 2 Anesthesiology, Unidade Local de Saúde da Região de Leiria, Leiria, PRT

**Keywords:** cytokine storm, dexamethasone, hlh, hlh associated with malignancy, hlh in adults, hscore, hyperinflammation

## Abstract

Hemophagocytic lymphohistiocytosis (HLH) is a severe hyperinflammatory syndrome characterized by uncontrolled immune activation, often associated with malignancy in adults. Early recognition and prompt initiation of treatment are crucial in managing this condition due to its rapid progression and poor prognosis.

We report the case of a 75-year-old male with suspected lymphoproliferative disease who presented with clinical criteria compatible with HLH. Immediate treatment with dexamethasone led to rapid clinical improvement, and the patient was referred to hematology for continued management of the underlying disease.

This case highlights the importance of constant clinical surveillance, early intervention, and the use of well-structured diagnostic tools in managing this syndrome in adults, where significant diagnostic and treatment challenges remain.

## Introduction

Hemophagocytic lymphohistiocytosis (HLH) is a life-threatening hyperinflammatory disorder characterized by uncontrolled activation of the immune system, resulting in organ damage [1]. The incidence of HLH is approximately 1.2 per 1,000,000 individuals annually worldwide [2]. This clinical condition may manifest as primary HLH (pHLH), typically presenting in children as a result of genetic mutations and characterized by impaired cytotoxicity, or as secondary HLH (sHLH), triggered by highly immunogenic factors [3,4].

In secondary HLH, the pathogenesis reflects the immune system’s inability to adequately control the effects of various triggers. Common causes of secondary HLH include infections (particularly viral infections such as EBV), autoimmune diseases, and malignancies. When associated with malignancy (mHLH), survival is extremely low (<20% at one year) [4,5]. The pathogenesis of mHLH is characterized by severe inflammation, persistent antigen stimulation by tumor cells, and loss of immune homeostasis due to chemotherapy, hematopoietic stem cell transplantation, or infection [6].

Early diagnosis and rapid therapeutic intervention are key in HLH, especially in adults, where it may present with unexpected manifestations such as fever, sepsis, hyperferritinemia, and bi/pancytopenia [7]. Although widely used diagnostic criteria, such as HLH-2004 (the 2004 revision by the Histiocyte Society for Hemophagocytic Lymphohistiocytosis), are effective, some time-consuming laboratory tests may not be feasible due to the syndrome’s rapid progression. Early recognition and prompt diagnosis are essential to facilitate timely therapeutic intervention and prevent rapid progression to organ failure and death [8].

Several malignancies are associated with HLH in adults, including T-cell or natural killer (NK) cell lymphomas (35%), B-cell lymphomas (32%), leukemias (6%), Hodgkin lymphoma (6%), other hematological malignancies (14%), solid tumors (3%), and other unspecified neoplasms (3%) [9,10]. The treatment of HLH in adults primarily follows the HLH-94 protocol (the first standardized treatment protocol for HLH developed by the Histiocyte Society in 1994), which is mainly used in pediatrics and is based on corticosteroids, cyclosporine A (CSA), etoposide, and, in some cases, intrathecal therapy [11].

## Case presentation

A 75-year-old man, independent in daily activities, with a medical history of hypertension and dyslipidemia treated with losartan 100 mg once daily and atorvastatin 10 mg once daily, was referred to the emergency department with complaints of abdominal pain, constipation, asthenia, anorexia, and a 6 kg weight loss over the course of one month.

Upon admission, his vital signs were normal for his age, with a tympanic temperature of 36.2°C, normal blood pressure, heart rate, and respiratory rate. Bowel sounds were present, and the abdomen was flat and slightly tender to palpation in the epigastric and mesogastric regions.

**Figure 1:**
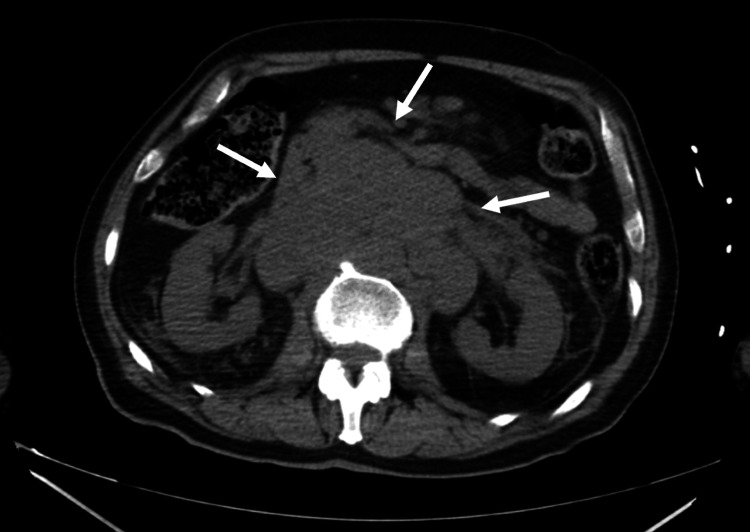
Axial abdominal CT showing a retroperitoneal lymphadenopathy conglomerate measuring 12 x 6 cm (white arrows). CT, computed tomography.

Laboratory results revealed leukocytes of 3.50 × 10³/µL, hemoglobin of 12.8 g/dL, platelets of 104 × 10³/µL, LDH of 2449 U/L, and CRP of 17.1 mg/dL (Table 1). Peripheral blood film showed the presence of three erythroblasts per 100 leukocytes. An abdominal CT scan showed a retroperitoneal lymphadenopathy conglomerate measuring 12 × 6 cm, with no compression of adjacent structures, and a normal-sized spleen and liver (Figure 1).

**Table 1:** Laboratory investigations CBC, complete blood count; WBC, white blood cells; LFT, liver function test; AST, aspartate transaminase; ALT, alanine transaminase; LDH, lactase dehydrogenase; CRP, C-reactive protein.

Test	Observed value at admission	72 Hours after admission	Reference range
CBC			
Hemoglobin	12.8 g/dL	10.6 g/dL	13.6–18.0
WBC	3.50 x 10³/μL	1.70 x 10³/μL	4.0–10.0
Platelets	104 x 10³/μL	68 x 10³/μL	140.0–440.0
Coagulation profile			
D-dimers	-	3953 ng/mL	<500
Fibrinogénio	-	327 mg/dL	200–393
LFT			
Total bilirubin	1.4 mg/dL	2.8 mg/dL	0.20–1.20
AST	14 U/L	181 U/L	5–34
ALT	12 U/L	47 U/L	0–55
Additional tests			
LDH	2449 U/L	5702 U/L	125–220
CRP	17.1 mg/dL	37.2 mg/dL	<0.5
Procalcitonin	-	0.18 ng/mL	<0.5
Ferritin	-	27,866 ng/mL	21.8–274.6
Triglycerides	-	312 mg/dL	<150

The patient was hospitalized for the investigation of a probable lymphoproliferative disorder. While awaiting a biopsy of the retroperitoneal lymphadenopathy, he developed a fever of 39.0°C, fatigue, and worsening analytical parameters (Table 1). The etiological investigation excluded viral and bacterial agents, including Epstein-Barr virus (EBV), human immunodeficiency virus (HIV), cytomegalovirus (CMV), hepatitis A, B, and C viruses (HAV, HBV, and HCV), varicella-zoster virus (VZV), and herpes simplex virus (HSV), with negative blood and urine cultures. As cytopenias and hyperferritinemia worsened, and given a high clinical suspicion of HLH, the Hscore (Table 2) was calculated at 180 points, corresponding to a 54%-70% probability for this condition [8].

**Table 2:** Parameters and points in the HScore *HIV-positive or receiving long-term immunosuppressive therapy (i.e., glucocorticoids, cyclosporine A, azathioprine). ^†^Defined as a hemoglobin level of 9.2 g/L and/or a leukocyte count ≤5 × 10^9^/L and/or a platelet count ≤110 × 10^9^/L. Source: Ref. [8].

Parameter	No. of points (criteria for scoring)
Known underlying immunosuppression*	0 (no) or 18 (yes)
Temperature (°C)	0 (<38.4), 33 (38.4–39.4), or 49 (>39.4)
Organomegaly	0 (no), 23 (hepatomegaly or splenomegaly), or 38 (hepatomegaly and splenomegaly)
No. of cytopenias^†^	0 (1 lineage), 24 (2 lineages), or 34 (3 lineages)
Ferritin (μg/L)	0 (<2000), 35 (2000-6000), or 50 (>6000)
Triglyceride (mmol/L)	0 (<1.5), 44 (1.5-4), or 64 (>4)
Fibrinogen (g/L)	0 (>2.5) or 30 (≤2.5)
Aspartate aminotransferase (U/L)	0 (<30) or 19 (≥30)
Hemophagocytosis on bone marrow aspirate	0 (no) or 35 (yes)

Given the urgency for treatment and the suspicion of a lymphoproliferative disorder, a CT-guided biopsy of the lymphadenopathy conglomerate was performed within 12 hours, and treatment was initiated with dexamethasone induction. The dosing regimen followed an eight-week induction schedule, starting with 8 mg twice daily for the first two weeks (10 mg/m² daily), followed by progressive tapering in the subsequent weeks, resulting in clinical and analytical improvement [11]. Subsequent histopathological analysis revealed a diagnosis of follicular non-Hodgkin lymphoma.

The patient had several risk factors for a poor prognosis, including male sex, presence of malignancy, advanced age, thrombocytopenia, hyperferritinemia, and leukopenia [7]. Despite the severe clinical presentation, he responded favorably to corticosteroid therapy, with significant clinical improvement within 48 hours of starting treatment. He was proposed for chemotherapy with R-CHOP, a regimen used to treat non-Hodgkin lymphoma consisting of rituximab, cyclophosphamide, doxorubicin, vincristine, and prednisone, and continued follow-up in the hematology outpatient clinic.

## Discussion

Although HLH is more common and best described in children, it also occurs in adults, where the most common triggers include malignancies, autoimmune diseases, and chronic infections, particularly viral ones such as EBV, CMV, VZV, HSV, and HIV [4]. Recently, treatments involving cell therapies have also been recognized to induce the condition [5].

The HLH-2004 diagnostic criteria (Table 3), initially developed for children, are often applied to adults, although they have not been validated for this age group [8]. Therefore, it is crucial to integrate these criteria with clinical judgment, especially in presentations where the severity of symptoms does not reflect the underlying disease status [12].

**Table 3:** HLH-2004 diagnostic criteria Source: Ref. [14]. HLH, hemophagocytic lymphohistiocytosis; NK cell, natural killer cell.

The diagnosis of HLH can be established if Criterion 1 or 2 is fulfilled
1. A molecular diagnosis consistent with HLH
2. Diagnostic criteria for HLH fulfilled (5 of the 8 criteria below)
Fever
Splenomegaly
Cytopenias (affecting ≥2 of 3 lineages in the peripheral blood)
Hemoglobin <90 g/L (hemoglobin <100 g/L in infants <4 wk)
Platelets <100 × 10^9^/L
Neutrophils <1.0 × 10^9^/L
Hypertriglyceridemia and/or hypofibrinogenemia
Fasting triglycerides ≥3.0 mmol/L (i.e., ≥265 mg/dL)
Fibrinogen ≤1.5 g/L
Hemophagocytosis in bone marrow or spleen or lymph nodes. No evidence of malignancy.
Low or no NK cell activity (according to local laboratory reference)
Ferritin ≥500 μg/L
sCD25 (i.e., soluble IL-2 receptor) ≥2400 U/mL

In adults, hyperferritinemia >10,000 µg/L has a sensitivity of 90% and a specificity of 96% for the diagnosis of HLH [13]. While hemophagocytosis can be observed in bone marrow biopsy, it is not an exclusive sign of HLH and is not considered a mandatory diagnostic criterion [14]. The sensitivity and specificity of hemophagocytosis in bone marrow examination for HLH are 83% and 60%, respectively. Therefore, a negative initial bone marrow specimen should not delay the diagnosis and initiation of HLH treatment [15].

Among the available tools for evaluation, the HScore has gained prominence for assessing the likelihood of HLH in adults, combining clinical and laboratory parameters [16,17]. It was developed based on the weighted parameters of HLH-2004, with an ideal cutoff value of 169 (sensitivity 93%, specificity 86%) [18]. In a subsequent study, the HScore demonstrated good diagnostic performance, particularly at initial presentation, although its accuracy decreases as the patient’s clinical status worsens [9].

Treatment in adults primarily follows the HLH-94 protocol, originally developed for pediatrics, which includes corticosteroids (dexamethasone), cyclosporine A (CSA), etoposide, and, in some cases, intrathecal therapy. In adults, especially older patients, dose adjustments of etoposide may be necessary, reducing frequency and/or dosage to avoid toxicity. Intrathecal therapy is only indicated in cases of progressive neurological symptoms or if cerebrospinal fluid (CSF) abnormalities persist after 2 weeks of treatment [8,19].

This case highlights several challenges in diagnosis and management. The report describes a patient admitted for the investigation of a possible lymphoproliferative disorder but without a definitive histopathological diagnosis at the time of cytokine storm, which delayed appropriate treatment of the underlying condition. Although an excisional lymph node biopsy is formally indicated, it was not performed in this patient due to the absence of palpable lymphadenopathy in easily accessible sites. The biopsy was eventually carried out after HLH was diagnosed, and treatment for HLH was initiated without a confirmed diagnosis of lymphoma. Nonetheless, it is important to emphasize that early treatment in this case was essential to prevent further complications and improve the patient’s prognosis.

The standard treatment for HLH includes corticosteroids and etoposide; however, this may limit chemotherapy due to the risks of severe adverse effects, such as myelosuppression and infections. When HLH is associated with lymphomas, the use of corticosteroids before chemotherapy has become routine, although secondary infections caused by corticosteroid use remain a significant cause of mortality, necessitating strict antimicrobial prophylaxis [3]. If there is an imminent risk of severe organ damage due to inflammatory lymphocytic proliferation, dose-adjusted etoposide can be considered to control HLH before the initiation of tumor-specific therapy. This may be combined with the CHOP (or CHOEP) protocol for lymphoma [8]. HLH treatment must be balanced with therapy for the underlying neoplasm, and in more severe cases, investigation of central nervous system involvement and even stem cell transplantation may be considered, depending on the type of lymphoma [19].

In recent decades, the recognition of HLH in adults has increased, but significant challenges remain, including the standardization of diagnostic criteria and the improvement of therapeutic strategies. While current treatments are effective, there is a clear need for new therapeutic options. Innovative drugs such as ruxolitinib, anakinra, alemtuzumab, and emapalumab are being investigated and used in specialized centers with the goal of improving the management of HLH and the prognosis of adult patients [11].

## Conclusions

HLH in adults is a complex condition with various triggers and genetic predispositions. Despite scientific advances, the prognosis remains poor, particularly when associated with malignancies, which are linked to significantly low survival rates. The applicability of the HLH-2004 criteria in adult patients remains debatable, as most evidence is derived from pediatric studies. Due to the lack of consensus in several areas, diagnostic and therapeutic algorithms must be applied cautiously and with clinical judgment. Although current therapeutic approaches are effective, new pharmacological options and specific diagnostic criteria are needed to improve disease management.
